# The Role of Transient Target Stimuli in a Steady-State Somatosensory Evoked Potential-Based Brain–Computer Interface Setup

**DOI:** 10.3389/fnins.2016.00152

**Published:** 2016-04-07

**Authors:** Christoph Pokorny, Christian Breitwieser, Gernot R. Müller-Putz

**Affiliations:** ^1^Laboratory of Brain-Computer Interfaces, Institute of Neural Engineering, Graz University of TechnologyGraz, Austria; ^2^Institute for Theoretical Computer Science, Graz University of TechnologyGraz, Austria

**Keywords:** brain–computer interface (BCI), steady-state somatosensory evoked potential (SSSEP), P300, electroencephalography (EEG), tactile stimulation, transient target stimulus

## Abstract

In earlier literature, so-called twitches were used to support a user in a steady-state somatosensory evoked potential (SSSEP) based brain–computer interface (BCI) to focus attention on the requested targets. Within this work, we investigate the impact of these transient target stimuli on SSSEPs in a real-life BCI setup. A hybrid BCI was designed which combines SSSEPs and P300 potentials evoked by twitches randomly embedded into the streams of tactile stimuli. The EEG of fourteen healthy subjects was recorded, while their left and right index fingers were simultaneously stimulated using frequencies selected in a screening procedure. The subjects were randomly instructed by a cue on a screen to focus attention on one or none of the fingers. Feature for SSSEPs and P300 potentials were extracted and classified using separately trained multi-class shrinkage LDA classifiers. Three-class classification accuracies significantly better than random could be reached by nine subjects using SSSEP features and by 12 subjects using P300 features respectively. The average classification accuracies were 48.6% using SSSEP and 50.7% using P300 features. By means of a Monte Carlo permutation test it could be shown that twitches have an attenuation effect on the SSSEP. Significant SSSEP blocking effects time-locked to twitch positions were found in seven subjects. Our findings suggest that the attempt to combine different types of stimulation signals like repetitive signals and twitches has a mutual influence on each other, which may be the main reason for the rather moderate BCI performance. This influence is originated at the level of stimulus generation but becomes apparent as physiological effect in the SSSEP. When designing a hybrid BCI based on SSSEPs and P300 potentials, one has to find an optimal tradeoff depending on the overall design goals or individual subjects' performance. Our results give therefore some new insights that may be useful for the successful design of hybrid BCIs.

## 1. Introduction

Brain–computer interfaces (BCIs) can provide a means of communication for persons who have lost all their motor control due to a severe neurological disease or brain injury (Wolpaw et al., 2002). In cases where the visual or auditory system is not functional, a BCI based on tactile stimuli might be the only way to provide a communication channel for such persons. One promising way to realize a tactile BCI is to use steady-state somatosensory evoked potentials (SSSEPs). SSSEPs can be evoked by repetitive tactile stimuli of sufficiently high rate (Regan, 1989). In healthy subjects, a two-class BCI based on SSSEPs could successfully be realized for the first time (Müller-Putz et al., 2006). On the one hand, users of such a BCI have to learn to focus attention on one of several stimulus locations, thereby modulating the respective SSSEP. On the other hand, the BCI needs to be trained to reliably detect such attention-modulated changes in the SSSEPs and translate them into an output channel for communication and control.

In several studies, attention modulation effects of SSSEPs and BCIs based on SSSEPs were investigated (Giabbiconi et al., 2004, 2007; Müller-Putz et al., 2006; Adler et al., 2009; Breitwieser et al., 2011; Severens et al., 2013; Pang and Mueller, 2014). In all of these studies, the use of some kind of randomly appearing transient target stimuli with increased or decreased amplitude which were embedded in the streams of repetitive tactile stimuli was reported. In our work, we follow the nomenclature of Müller-Putz et al. (2006) and refer to these transient target stimuli as “amplitude twitch” or simply “twitch.” Typically, the subjects were instructed to actively recognize (e.g., to silently count) these twitches, in order to force the subjects to focus maximal attention on the desired stimulation site. Otherwise, keeping attention focused on one of different simultaneous streams of stimuli over some period of time would be a virtually impossible task. In most of these studies, trials with twitches were included in the data analysis without treating them in a particular way (Giabbiconi et al., 2004, 2007; Müller-Putz et al., 2006; Breitwieser et al., 2011), whereas, Adler et al. (2009) and Pang and Mueller (2014) excluded them from further analyses in order to investigate pure SSSEPs. In the study of Severens et al. (2013), a successful attempt was made to explicitly include transient event-related potentials (ERPs) caused by twitches in the analyses and to directly compare the BCI performance using transient and steady-state responses for the first time.

However, in none of these studies, the effects of twitches on the stimulation signal and subsequently, on the SSSEP itself were explicitly analysed from a signal processing point of view in terms of intended or unintended temporal or spectral changes of the stimulation signal. Arguably, a transient change in the repetitive stimulation signal will have some impact on the SSSEP. Here, an important question is, if such effects are negligible or may cause some undesired physiological effects, such as degraded classification performance in a BCI. In some cases, as shown by Xu et al. (2013) in the context of steady-state visually evoked potentials (SSVEPs), seemingly negative effects may even be turned to some new kind of features (“blocking features") which can even be beneficial for classification.

The aim of our work is to ask this question again and to critically revisit the use of twitches in an SSSEP-based BCI. For this purpose, a hybrid BCI based on tactile stimuli was designed to throw some new light on the use of twitches. In general, the idea of a hybrid BCI is to combine different brain signals in a meaningful way in order to improve the performance and to make the BCI applicable to a broader range of subjects or patients (i.e., lower number of illiterates) (Pfurtscheller et al., 2010; Müller-Putz et al., 2015). Similar as Severens et al. (2013), we investigate in our study a BCI which combines SSSEPs and P300 potentials evoked by twitches embedded into the streams of tactile stimuli. However, these two brain signals are somehow mutually exclusive, since the former is a frequency-domain signal whereas the latter is a time-domain signal. According to the Fourier uncertainty principle (Gabor limit) a signal cannot be both time-limited and band-limited at the same time (Gabor, 1946). We therefore investigate the role of twitches in the context of SSSEPs and address the questions if SSSEPs and P300 potentials can be evoked concurrently and under what conditions the performance of a hybrid BCI may be improved by combining SSSEPs and P300 potentials.

## 2. Methods

The impact of twitches on SSSEPs was investigated in a real-world BCI setup. For this purpose, we designed an online BCI following the standards for open interfaces for communication (TiA, TiD) described by Müller-Putz et al. (2011). For the online BCI and all offline analyses, Matlab/Simulink (The MathWorks, Inc., MA, USA) together with the EEGLAB (Delorme and Makeig, 2004) and BCILAB (Kothe and Makeig, 2013) toolboxes were used.

### 2.1. Experimental paradigms

Within this study, EEG (electroencephalogram) measurements were conducted in three successive parts using experimental paradigms for (i) EOG (electrooculogram) recording needed for EOG artifact removal, (ii) screening for subject-specific “resonance-like” frequencies of the somatosensory system (Müller et al., 2001), and (iii) a cue-based online BCI paradigm.

#### 2.1.1. EOG recording

The first part of each measurement was to record 2 min of induced EOG artifacts. Following the procedure described by Schlögl et al. (2007), each subject was instructed to perform 1 min of eye movements and 1 min of blinking only. Using this recording, parameters for an autoregressive model were estimated which was then used to automatically remove EOG artifacts from online BCI recordings.

#### 2.1.2. Screening

As demonstrated in various experiments in literature, each person shows a characteristic tuning curve and reacts with specific “resonance-like” frequencies of the somatosensory system in response to repetitive tactile stimuli (Tobimatsu et al., 1999, 2000; Müller et al., 2001; Breitwieser et al., 2012). “Resonance-like” frequencies are frequencies with maximal SSSEP amplitude and reflect resonance phenomena of the underlying frequency-selective neuronal networks. In SSSEP-based BCIs, the use of subject-specific stimulation frequencies with strongest SSSEP responses is assumed to yield higher BCI performance compared to when using the same standard frequencies for all subjects (Müller-Putz et al., 2006; Breitwieser et al., 2011; Severens et al., 2013). Therefore, a screening procedure similar as described by Breitwieser et al. (2012) was applied to identify these subject-specific “resonance-like” frequencies, and to select two frequencies for left and right index finger stimulation to be used in the subsequent BCI paradigm.

**Figure 1:**
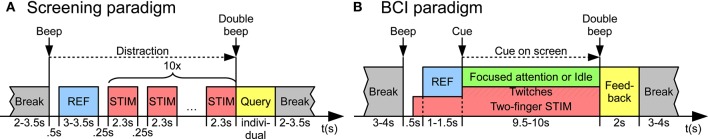
**Experimental paradigms**. **(A)** In the screening paradigm, each trial started with a reference (REF) period, followed by 10 stimulation (STIM) intervals with frequency and index finger randomly chosen. Subjects had to perform a distracting mental arithmetic task during the whole trial. At the end of each trial, they were asked for their calculation results. **(B)** In the online BCI paradigm, both index fingers were simultaneously stimulated. Each trial started with a reference period, followed by a focused attention or idle period, as indicated by a cue appearing on the screen. Twitches were only presented during this period (hatched area). At the end of each trial, a discrete feedback was given on the screen.

The left and right index finger tips were randomly stimulated with 10 frequencies ranging from 17 to 35 Hz in steps of 2 Hz. As shown in Figure 1A, each trial started with a reference period (length 3–3.5 s) without stimulation, followed by 10 stimulation intervals (length 2.3 s; only the last 2 s were used for analysis). In each stimulation interval, frequency and index finger were randomly chosen. The only restriction was that the exact same frequency and finger could not be selected twice in succession. Short pauses (length 0.25 s) were added between different stimulation intervals. To avoid attention modulation effects during screening, the subjects were not supposed to focus attention on the stimuli. Therefore, subjects had to perform a distracting mental arithmetic task during the whole trial (Breitwieser et al., 2011). They had to continuously add or subtract random numbers appearing on the screen in front of them. At the end of each trial, they were asked for their calculation results in order to monitor their distraction. The whole screening was divided into eight runs with 10 trials each. In total, 40 repetitions per frequency and index finger were recorded, resulting in a total amount of 800 repetitions per subject.

After screening, tuning curve maps showing the percentage band power increase of the stimulation intervals relative to the reference intervals (Müller et al., 2001) were computed for seven bipolar channels above the somatosensory cortex. Two stimulation frequencies with the highest and most similar responses in the tuning curve maps were manually selected for each subjects. The only restriction was that the selected frequencies had to be separated by at least one other stimulation frequency in between. The selected frequencies were then used in the subsequent BCI paradigm for left and right index finger stimulation.

#### 2.1.3. BCI paradigm

The left and right index fingers were simultaneously stimulated using the two frequencies selected after the screening procedure. The subjects were randomly instructed by a cue on the screen to focus attention on one or none of the fingers. The target finger was indicated by an arrow pointing to the left (“Focus left” class) or right (“Focus right” class). In one third of all trials, no arrow was shown and the subjects were instructed to avoid focusing attention on any finger (“Idle” class). Since the repetitive stimulation signals are usually just perceived as continuous vibrations, it is generally very difficult to focus attention and keep attention focused on the target finger. In order to make the focusing attention task easier, short twitches were inserted in the stimulation patterns of both fingers at random time points (see Section 2.2 for more details). Such twitches were short interruptions in the repetitive stimulation signals and could be perceived as rare, discrete events in the repetitive streams of stimuli. So, to keep attention focused on the target finger, the subjects were instructed to actively recognize and silently count the twitches appearing on the target, and to ignore twitches on the non-target finger. During the whole trial, the subjects were also instructed to avoid shifting their gaze and to just look at the center of the screen indicated by a cross.

As shown in Figure 1B, each trial started with a beep tone and the cross appearing on the screen. After 0.2 s, the stimulation of the left and right index fingers started. After a waiting time of 0.5 s after trial start, there was a reference period with a random length between 1 and 1.5 s without focused attention, where the subjects just had to look at the cross on the screen. Then, an arrow faded in on the screen instructing the subjects on which finger to focus attention on, or no arrow appeared for idle trials. The length of such a focus attention or idle period respectively was randomly chosen between 9.5 and 10 s. Twitches were presented only during this period and not during the reference interval. Each trial ended with a double beep followed by a discrete feedback appearing for 2 s on the screen. The feedback indicated if the target class was correctly detected (green circle), wrongly detected (red circle), or if no decision could be made (yellow circle). After the feedback, a random break between 3 and 4 s was added before the start of the next trial. Class decisions were made by two combined classifiers, one for SSSEPs and one for P300 potentials evoked by twitches (see Section 2.5 for details). The whole online BCI paradigm was divided into eight runs with 10 trials per class each. The following classifier update strategy was chosen: During the first two runs, data were just recorded and no feedback was given. After two runs, the classifiers were trained based on data from the first two runs and used to provide feedback in the following two runs. After four runs, the classifiers were retrained again based on data from all four previous runs and used to provide feedback in the remaining four runs of the measurement. The data from all eight runs (80 trials per class) were altogether used in the offline analyses.

### 2.2. Tactile stimulation

**Figure 2:**
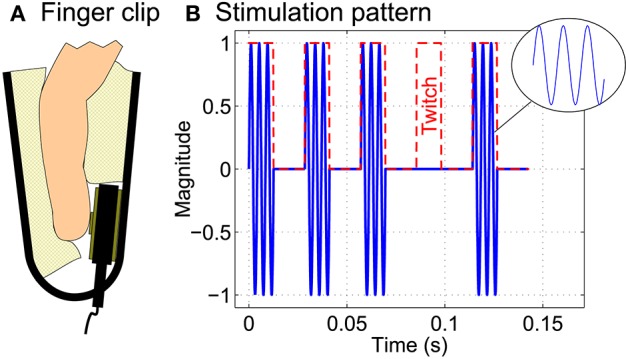
**Tactile stimulation**. **(A)** Two C-2 tactors were attached to the left and right index finger using finger clips. **(B)** The tactile stimulation pattern consisted of a 237 Hz sinusoidal carrier signal which was amplitude modulated with a rectangular stimulation signal (red dashed line) of the respective stimulation frequency (here: 35 Hz). Twitches were implemented as complete interruption of the stimulation signal for exactly one stimulation period.

Two C-2 tactors (Engineering Acoustics, Inc., Casselberry, Florida, USA) were attached to the left and right index finger. In order to have constant contact pressure betweeen tactors and fingers, finger clips as depicted in Figure 2A were used for this purpose. The prototype of a self-made tactile stimulation device (Pokorny et al., 2014) was used to generate the complex repetitive and transient stimulation patterns needed to evoke SSSEPs as well as P300 potentials. The stimulation pattern consisted of a 237 Hz sinusoidal carrier signal which was amplitude modulated with a rectangular signal of the respective stimulation frequency (see Figure 2B), similar as used in previous studies involving SSSEPs (Müller-Putz et al., 2006; Breitwieser et al., 2011, 2012; Pokorny et al., 2014). The duty cycle was chosen close to 50% in such a way that the carrier signal always started and stopped at phase zero.

During the online BCI runs, seven twitches per finger and trial were pseudo-randomly embedded into the stimulation patterns. As visible in Figure 2B, twitches were implemented as complete interruption (100% attenuation) of the repetitive stimulation signal for exactly one period of the respective stimulation frequency. This was done to have a strong and clearly defined effect when explicitly investigating the role of twitches. In contrast, in previous studies, e.g., by Breitwieser et al. (2011) and some pilot studies (unpublished), we realized twitches only as moderate attenuation of the stimulation signal. However, in those studies, twitches were generally reported by the subjects to be hardly perceivable and almost impossible to recognize or count.

**Figure 3:**
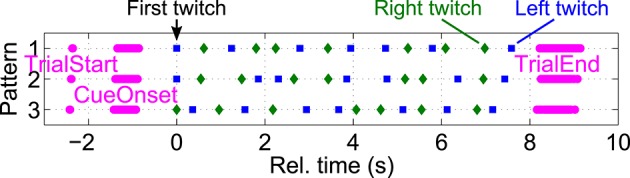
**Pseudo-randomized twitch patterns**. To ensure that twitches would not occur closer than 250 ms, three different randomized twitch patterns were generated beforehand for all possible stimulation frequency combinations (here: 33 and 27 Hz). In each trial, one of these three pattern was randomly chosen. Left (blue squares) and right (green diamonds) twitch positions, as well as trial start, cue onset, and trial end (magenta bullets) are shown for individual trials, aligned to the first twitch's positions (*t* = 0 s).

Twitch positions were distributed in such a way that they would occur as rare, random events, suitable to evoke P300 potentials when the subjects actively focus attention on them. To ensure that twitches would not occur too close after each other within and across hands, three different randomized twitch patterns were generated beforehand for all possible combinations of left and right stimulation frequencies. In each trial, one of these three pattern was randomly chosen. The minimal inter-stimulus interval between consecutive twitches at the same hand and across hands was 250 ms each. Twitch onsets were precisely (in the order of μs) recorded by means of two additional (optically isolated) trigger channels from the stimulation device to the EEG amplifier. Figure 3 shows an example of three different twitch patterns generated for stimulation frequencies of 33 and 27 Hz respectively. Twitch positions, as well as trial start, cue onset, and trial end positions are shown as separate markers for individual trials. All positions within trials were aligned to the corresponding first twitch's positions. Due to random reference and focus attention period lengths, this results in variable trial start, cue onset, and trial end positions relative to the first twitch.

### 2.3. Participants

Fifteen healthy subjects voluntarily participated in this study. They were paid for participation and were informed in detail about the aims of this study. None of them reported any neurological disease. All subjects gave written informed consent, and the study was conducted in accordance with the local ethics regulations (Medical University Graz) and the Declaration of Helsinki. The measurement of one subject was aborted since he or she did not follow the instructions given by the experimenter. The remaining fourteen subjects (seven male/female) were aged between 20 and 39 years [mean 26.3 ± 6.2(SD) years].

### 2.4. EEG recording

**Figure 4:**
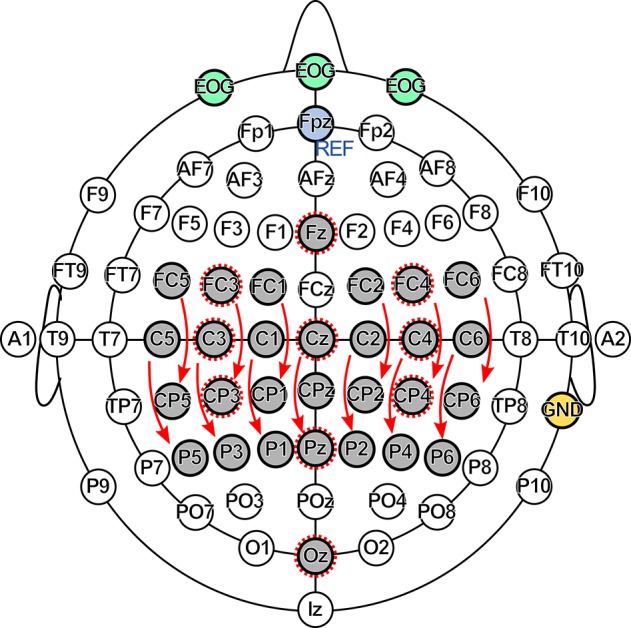
**Electrode setup**. The EEG was recorded at 29 channels (gray), the EOG at 3 channels (green). The Fpz electrode (blue) was used as reference (REF), the right mastoid (yellow) as ground (GND). The SSSEP channel set consisted of 13 bipolar channels indicated by arrows, the P300 channel set of 10 monopolar channels indicated by dashed circles.

The EEG was recorded from 29 channels together with 3 EOG channels, as shown in Figure 4. The channel Fpz was used as reference, the right mastoid as ground. Data were recorded using two g.USBamp biosignal amplifiers (g.tec medical engineering GmbH, Austria) with active electrodes and a sampling rate of 600 Hz. A bandpass filter between 0.5 and 200 Hz, and a notch filter at 50 Hz were applied. All measurements were conducted in a shielded room. The subjects were seated in an armchair in front of a computer screen with their hands comfortably placed on armrests during the measurements.

For SSSEP investigations, 13 bipolar channels above the somatosensory cortex indicated by arrows in Figure 4 were preselected and are referred to as SSSEP channel set. Similarly, 10 monopolar channels indicated by dashed circles in Figure 4 were preselected for investigating P300 effects and are referred to as P300 channel set.

### 2.5. Data analysis

#### 2.5.1. Artifact removal

EEG channels with obviously bad signal quality found after visual inspection of the EEG signals during measurements were excluded from data analysis (five channels in total). EOG artifacts were removed based on autoregressive parameters estimated from the EOG recording (Schlögl et al., 2007). Trials contaminated with EMG (electromyogram) and other types of strong artifacts were removed using a simple theshold-based artifact detection method (Delorme et al., 2007). Artifact thresholds of 90 and 60 μV were used for monopolar and bipolar channels respectively.

#### 2.5.2. Brain–computer interface

Feature for SSSEPs and P300 potentials were extracted and classified using separately trained classifiers. For SSSEPs, logarithmic lock-in amplifier system (LAS) features (Müller-Putz et al., 2006) were extracted from the 13 bipolar channels from the SSSEP channel set. A filter bandwidth of 2 Hz around each stimulation frequency was used and a moving average (MAV) filter with 1 s length was applied. The mean SSSEP strength was estimated by averaging the LAS features over time within an interval from 1 to 8.5 s after cue onset and used for classification.

For P300 potentials, the 10 monopolar channels from the P300 channel set were selected and low-pass filtered using a 3rd-order Butterworth filter at 10 Hz. Time segments from 0 to 800 ms after each twitch onset (read out from trigger channels) were extracted, resulting in seven twitch segments for each of the index fingers per trial. The seven segments of each finger were linearly detrended, averaged, downsampled by a factor of ten, and used for classification. The influence of the number of averages on the P300 performance was separately investigated by using different numbers of averages ranging from one to seven. For numbers of twitches lower than seven, random subsampling was used to select subsets of twitches within trials. The whole procedure was repeated 10 times in order to get a reliable estimate.

For both types of features, multi-class shrinkage LDA classifiers (Schäfer and Strimmer, 2005) based on the one-vs-all strategy were used to predict the target class. The overall BCI performance was estimated using 10 × 10 cross-validation to avoid overfitting. To identify classification results that were significantly better than a random classifier, we compared our results to the real chance level (Müller-Putz et al., 2008) instead of to the theoretical one (33.3%). The real chance level takes a confidence interval at significance level α = 1% into account and was computed based on the total number of trials per class to be 40.8%. So all classification results exceeding this level can be regarded as significantly better (at α = 1%) than just random results. For online feedback presentation, the decisions from both classifiers were combined in order to arrive at a final decision. Together with the class prediction, each classifier returned a linear score which was mapped to a class probability value, representing a measure of certainty (0–100%) about their decisions. The result from the classifier with the higher probability value was selected as final decision. If none of the classifiers reached a probability threshold of at least 50%, no decision was made.

#### 2.5.3. Effects of twitches on the SSSEP

To investigate the effects of twitches on the SSSEP, different visualization and analysis methods were implemented. A time-frequency representation was chosen which is capable of visualizing steady-state as well as transient signals. For this purpose, spectrograms were computed based on the short-time Fourier transform (STFT) showing the power spectral densities (PSD) at different frequencies over time. The STFTs were computed using a 4096-point fast Fourier transform (FFT). As already mentioned (see Section 2.2), in each trial, one of three pseudo-randomized twitch patterns was randomly chosen. Separate spectrograms were computed by averaging the PSDs of all trials belonging to the same twitch pattern. Before averaging, the corresponding time axes were aligned either to the trial start or first twitch's position as needed. Since spectrograms are subject to the Fourier uncertainty principle, the choice of the window has a strong impact on their time-frequency resolution. Long time windows result in better frequency but poor time resolution, whereas short time windows result in good time but poor frequency resolution. Minimizing the Fourier uncertainty principle, the best simultaneous time-frequency resolution can be achieved with a Gaussian window function (Gabor, 1946) which was used in our analysis. Gaussian windows with two different lengths were applied: (i) To visualize the (steady-state) frequency content, a fixed window length of 3 s and an overlap between consecutive window segments of 2.95 s was chosen. (ii) To visualize transient effects, a fixed window length of 1 s and a window overlap of 0.95 s was chosen.

**Figure 5:**
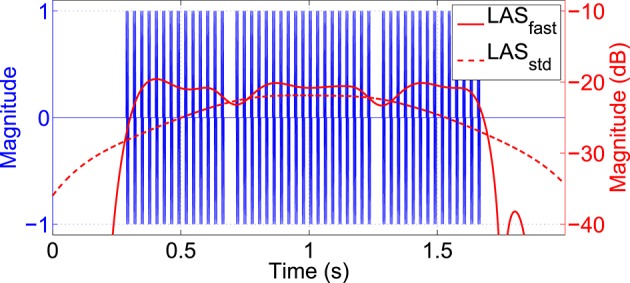
**Effect of twitches in an ideally simulated 35 Hz stimulation signal**. Logarithmic LAS features were extracted at the stimulation frequency using a fast setting (4 Hz bandwidth, no MAV filter; red solid line) or standard setting (2 Hz bandwidth, 1 s MAV filter; red dashed line). With the fast setting, effects of twitches are clearly visible as attenuations of the LAS amplitude at twitch locations.

To specifically reveal potential transient effects of twitches on the SSSEP, the idea was to extract LAS features from both stimulation frequencies and test them for significant changes (decreases) at twitch locations. Since the settling time of the LAS is inverse proportional to its bandwidth, a bandwidth of 4 Hz around each stimulation frequency was chosen for this purpose. Additionally, the MAV filter was omitted, resulting in a much faster LAS setting. Figure 5 shows an example of how this method is capable of extracting short transient disruptions caused by twitches from an ideally simulated stimulation signal with 35 Hz. Effects of twitches are clearly visible as attenuations of the LAS amplitude at the twitch locations. In comparison, LAS features extracted with the standard setting (2 Hz bandwidth, 1 s MAV filter) are not capturing such transients.

To test if such attenuations caused by twitches are not only present in the stimulation signal but also in the resulting SSSEPs (referred to as SSSEP blocking) a Monte Carlo permutation test (Nichols and Holmes, 2001) was applied. Since this is a non-parametric approach, no assumptions about the distribution of values being tested were required. The permutation test was based on the null hypothesis that no significant decrease in SSSEP amplitude caused by twitches was present and therefore, twitch pattern labels (i.e., fixed twitch positions within each trial) would be interchangeable across trials. So, twitch patterns were randomly permuted, changing the assignment of twitch patterns to trials, without changing the overall distribution of twitch positions. As test statistic, the average SSSEP amplitude over all trials and twitches extracted from 50 ms time intervals around twitch onsets (positions according to real or permuted twitch pattern assignment) was computed. In this way, a null distribution of average SSSEP amplitude values was generated based on the real assignment and *N* = 100, 000 permutations. The false positive probability (FPP) that an observed decrease in SSSEP amplitude is just a random effect was estimated as percentage of values in the null distribution that were lower than or equal the actually observed one. Since the actually observed value was always part of the null distribution, the resulting FPP could never be smaller than 1∕(*N*+1). The FPP was estimated in steps of 50 ms from −500 to +500 ms around twitch onsets, in order to identify intervals of significant blocking effects. An FPP below some significance level α was then considered as significant SSSEP blocking effect. An α-level of 1%, Bonferroni corrected for multiple testing based on the number of time intervals, stimulated fingers, and observed channels was applied. Only two bipolar channels above the somatosensory cortex (FC3-CP3 and FC4-CP4), where the highest SSSEP responses and therefore, the strongest effects were expected, were included in this significance test.

This significance test was intended to identify significant SSSEP blocking effects disregarding any class information. As a next step, we investigated if potential SSSEP blocking effects were modulated by attention and could, therefore, be beneficial features for classification, similar as shown by Xu et al. (2013) in the context of SSVEPs. For this purpose, we extracted SSSEP features at both stimulation frequencies using the fast LAS setting from intervals of 0 to 400 ms after twitch onsets. Segments from all seven twitches per finger and trial were averaged over time for different channels and used as blocking features for classification. All 13 bipolar channels from the SSSEP channel set were used for this purpose. Again, a multi-class shrinkage LDA classifier together with 10 × 10 cross-validation was applied for performance estimation.

## 3. Results

### 3.1. Steady-state somatosensory evoked potentials

**Figure 6:**
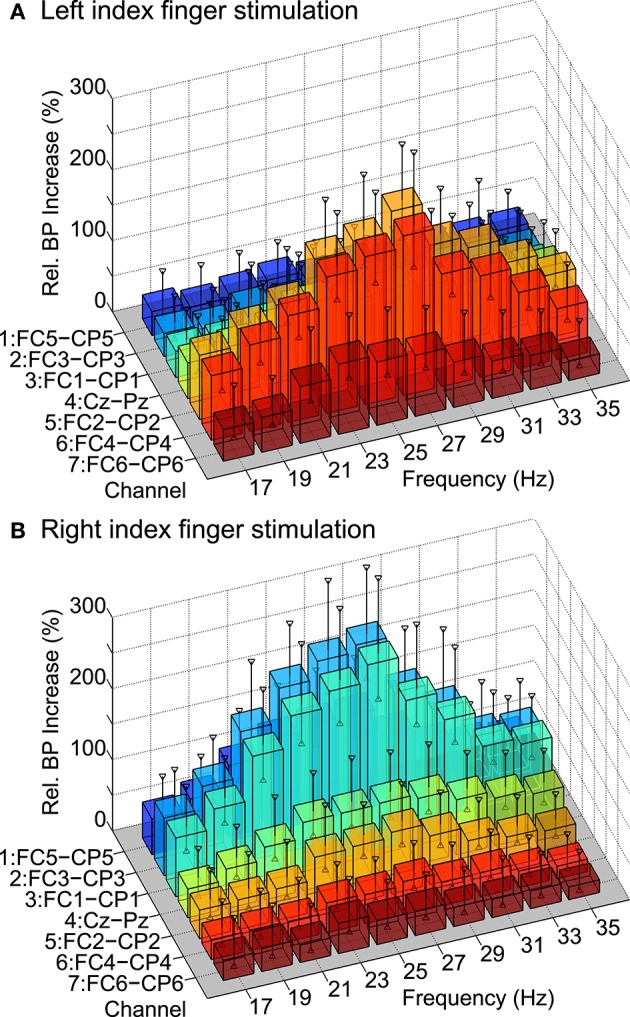
**Grand average tuning curve maps over all subjects**. The relative bandpower increase is shown at 10 stimulation frequencies and seven bipolar channels above the somatosensory cortex for **(A)** left and **(B)** right index finger stimulation. Vertical markers indicate grand average confidence intervals (α = 95%) estimated with bootstrapping.

SSSEP responses could be found in all subjects after screening for subject-specific “resonance-like” frequencies of the somatosensory system. In Figure 6, the grand average tuning curve maps over all subjects obtained after the screening procedure can be found. The relative bandpower increase is shown at 10 stimulation frequencies and seven bipolar channels above the somatosensory cortex for left and right index finger stimulation. Vertical markers indicate the grand average of the 95% confidence intervals estimated with bootstrapping based on 1000 bootstrap samples. As expected, the tuning curve maps show largest bandpower increases at channels contralateral to the stimulated finger.

Table 1A summarizes the individual screening results of all subjects. The manually selected stimulation frequencies for left and right index finger stimulation to be used in the BCI paradigm are shown, which were in the range of 21 to 35 Hz. Additionally, the relative bandpower increases at bipolar channels contralateral to the stimulated hand, namely FC4-CP4 for left and FC3-CP3 for right index finger stimulation can be found. Relative bandpower increases at these channels were in the range of 53–536%. On average, relative bandpower increases of 235 ± 133(SD)% for left and 269 ± 127(SD)% for right index finger stimulation could be found.

**Table 1:** **Summary of individual screening, blocking interval estimation, and classification results**.

**Code**	**(A) SSSEP screening**				**(B) SSSEP blocking intervals**		**(C) Performance**			
	***f*_*L*_ (Hz)**	***f*_*R*_ (Hz)**	***relBP*_*L, c*_ (%)**	***relBP*_*R, c*_ (%)**	***BLInt*_*L, c*_ (ms)**	***BLInt*_*R, c*_ (ms)**	**SSSEP (%)**	**P300 (%)**	**BLFtr (%)**	**Combined (%)**
s01	25	21	536	475	50–150	150–200	**47.1**	**47.3**	29.8	38.2
s02	23	27	356	482	–	100–150	**56.1**	**51.1**	**49.4**	**50.9**
s03	33	27	114	133	–	–	**45.5**	**58.1**	**46.0**	**64.3**
s04	31	35	291	288	50–150	–	39.5	**41.9**	35.7	39.8
s05	25	29	190	201	–	–	36.0	**45.7**	38.9	**47.8**
s06	27	23	291	315	200–250	–	**42.7**	**44.2**	**46.5**	**50.1**
s07	23	27	161	419	–	–	**46.0**	**42.3**	38.7	40.5
s08	33	27	376	291	50–100	50–100	**46.5**	39.2	**44.8**	**46.6**
s09	25	29	155	165	–	–	31.4	**42.4**	23.3	24.0
s10	29	25	53	101	–	–	9.2	36.0	16.0	10.8
s11	23	27	234	294	50–200	50–100	**44.4**	**65.5**	38.6	33.0
s12	23	27	120	123	–	–	**47.5**	**64.1**	**46.4**	**66.2**
s13	27	21	310	308	50–150	100–250	**61.8**	**57.5**	**44.2**	**62.9**
s14	21	25	101	165	–	–	39.5	**48.1**	32.6	33.6
Mean			235	269			48.6	50.7	46.2	55.5
SD			133	127			6.2	8.5	1.8	8.5

***(A)** SSSEP screening: Manually selected stimulation frequencies for left (f_L_) and right (f_R_) index finger stimulation; Relative bandpower increases at contralateral bipolar channels, FC4-CP4 for left (relBP_L, c_) and FC3-CP3 for right (relBP_R, c_) hand stimulation. **(B)** SSSEP blocking intervals: Significant blocking intervals (BLInt) for left and right index finger stimulation at the bipolar channels FC3-CP3 and FC4-CP4 contralateral to the stimulated hand. **(C)** Performance: Cross-validated BCI performance using SSSEP, P300, and blocking features (BLFtr) separately or combined for classification. Mean and SD were computed only over subjects with accuracies significantly better than random [40.8% at α = 1% (Müller-Putz et al., 2008); bold values]*.

**Figure 7:**
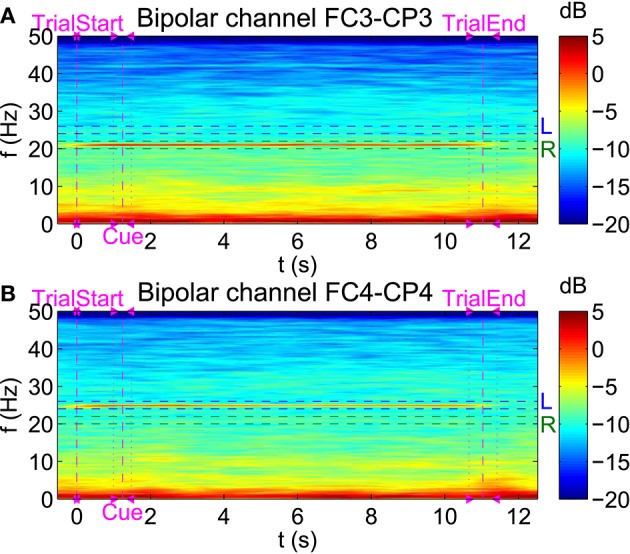
**Visualization of SSSEPs**. Spectrograms (4096-point FFT, 3 s window length, 2.95 s window overlap) of subject s01 showing the PSD (in dB) during simultaneous left (25 Hz) and right (21 Hz) index finger stimulation can be seen for the bipolar channels **(A)** FC3-CP3 and **(B)** FC4-CP4.

To visualize SSSEPs at the selected stimulation frequencies during the online BCI runs, spectrograms with a window length of 3 s and an overlap between consecutive window segments of 2.95 s were computed. As an example, in Figure 7, spectrograms of subject s01 showing the PSD during simultaneous left and right index finger stimulation with 25 and 21 Hz respectively can be found. Spectrograms are shown for the bipolar channels FC3-CP3 and FC4-CP4 during the course of a trial. Trials belonging to only one of the three twitch patterns were averaged, and individual trials were aligned to each trial's start position. SSSEPs are clearly visible at the respective channel contralateral to the stimulated hand.

### 3.2. P300 potentials

**Figure 8:**
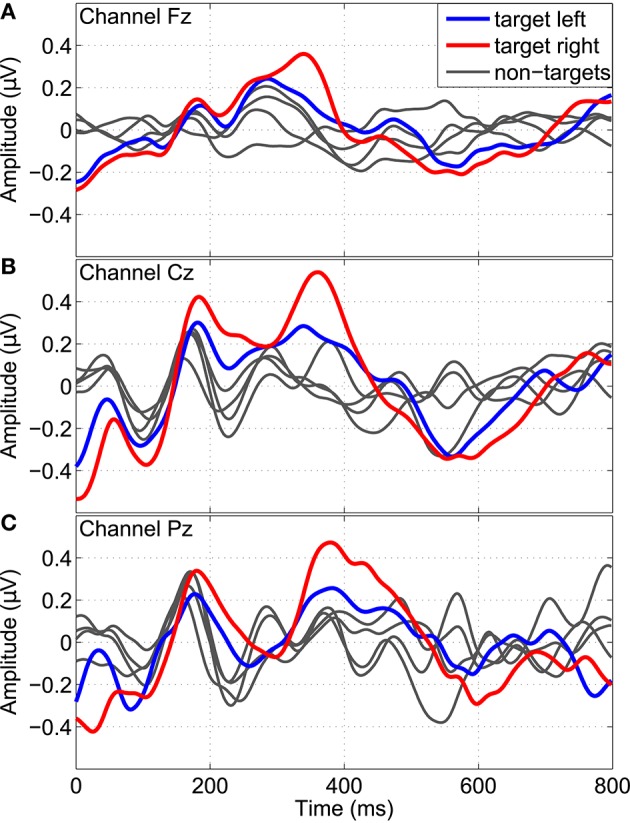
**P300 response evoked by twitches randomly embedded into the stream of repetitive stimuli**. Grand average time segments (0–800 ms) after twitch onsets over all subjects are shown for the channels Fz, Cz, and Pz **(A–C)**. Separate segments are shown for left (bold blue) and right (bold red) target twitches, as well as for all remaining non-target conditions (gray).

By embedding twitches at random positions into the streams of repetitive tactile stimuli, P300 potentials could be evoked. Figure 8 shows the grand average P300 response over all subjects divided into different twitch locations (left or right hand) and target classes (left cue, right cue, or idle cue). Averaged time segments from 0 to 800 ms after twitch onsets are shown for the monopolar channels Fz, Cz, and Pz. A P300 response can be seen around 300–400 ms in response to left and right target twitches (i.e., at left twitch locations for left classes and at right twitch locations for right classes), most pronounced at channel Cz. After non-target twitches, no clear P300 potentials can be found.

**Figure 9:**
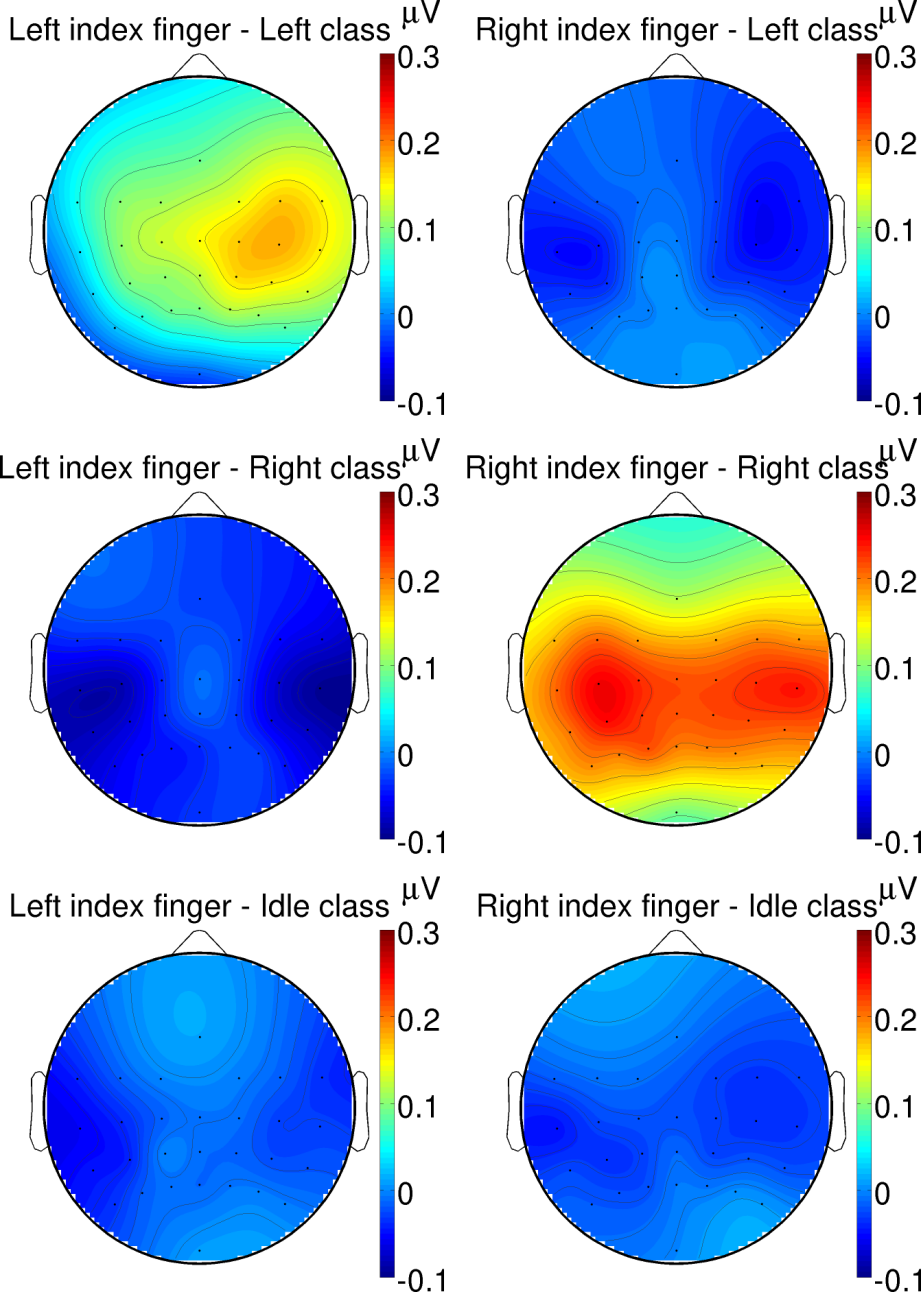
**Grand average topographic plots of the P300 response**. The spatial distribution of the P300 component extracted from a 200–500 ms time window is shown for different twitch locations (left/right column: left/right hand) and target classes (from top to bottom: left/right/idle cue).

Additionally, in Figure 9, grand average topographic plots show the spatial distribution over all subjects of the P300 component extracted from a 200–500 ms time window. It can be seen that the P300 component is most prominent after target twitches at central channel location above the somatosensory cortex. For the left target twitches, a shift toward contralateral channels can be observed while for right target twitches, a bilateral activation can be found.

### 3.3. Effects of twitches on the SSSEP

**Figure 10:**
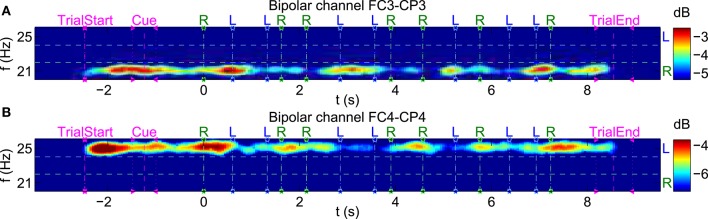
**Visualization of transient effects in the SSSEP**. Spectrograms for the bipolar channels **(A)** FC3-CP3 and **(B)** FC4-CP4 of subject s01 as in Figure 7, but with shorter time windows (1 s window length, 0.95 s window overlap) and individual trials aligned to each first twitch's position. The color axes were scaled to highlight variations at each stimulation frequency. Twitch onsets at left (L) and right (R) index fingers are drawn as vertical dashed lines.

To visualize transient effects in the SSSEP, spectrograms with a window length of 1 s and a window overlap of 0.95 s were computed. As an example, Figure 10 shows spectrograms for the same subject and trials as in Figure 7. This time, individual trials were aligned to each corresponding first twitch's position, and positions of all twitch onsets are drawn in the spectrograms. Moreover, the color axes were individually scaled to highlight PSD variations over time at the stimulation frequencies. Interestingly, SSSEP blocking effects, namely an attenuation of roughly 2–3 dB of the SSSEPs time-locked to the corresponding twitch onsets can be observed. In more detail, attenuations at the left stimulation frequency seem to be time-locked to left-hand twitches (visible at channel FC4-CP4). Similarly, attenuations at the right stimulation frequency seem to be time-locked to right-hand twitches (visible at channel FC3-CP3).

**Figure 11:**
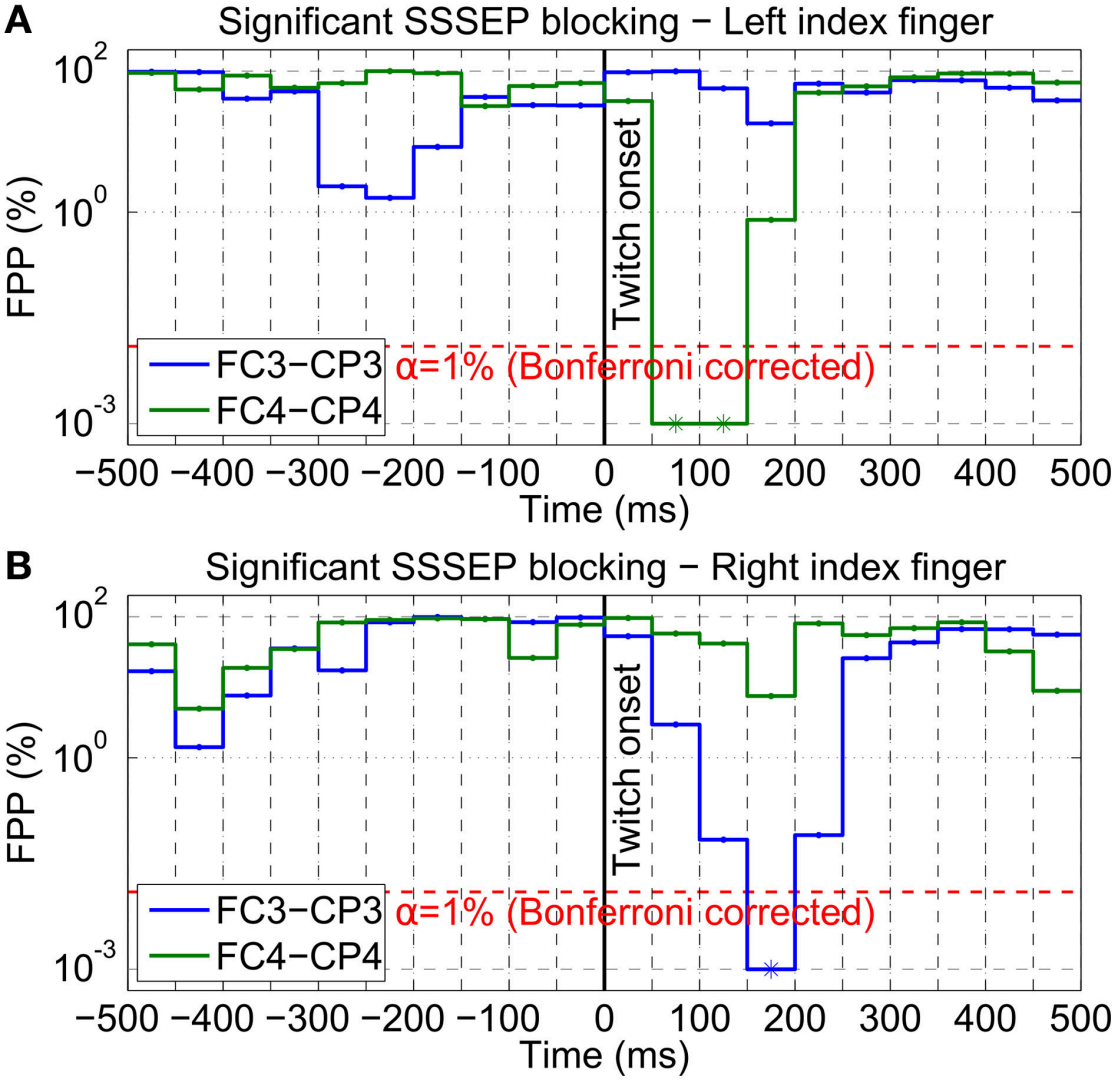
**Blocking interval estimation of subject s01**. SSSEP blocking effects of **(A)** left-hand and **(B)** right-hand twitches were statistically validated using a permutation test (100, 000 repetitions; 4 Hz LAS bandwidth; no MAV filter). The FPP (in %; log y-axis) was estimated in steps of 50 ms from −500 to +500 ms around twitch onsets for two bipolar channels (blue: FC3-CP3; green: FC4-CP4). Significant blocking effects (indicated by asterisks) can be found in intervals where the FPP is below the indicated significance level (α = 1%; Bonferroni corrected; red dashed line).

To statistically validate this visual impression, significant blocking time intervals were determined by means of a permutation test based on SSSEP amplitudes extracted with the fast LAS setting. As an example, Figure 11 shows the estimated FPPs (i.e., the probabilities that the observed decreases in SSSEP amplitude are random effects) for subject s01 at the bipolar channels FC3-CP3 and FC4-CP4 in the interval from −500 to +500 ms around twitch onsets for left-hand and right-hand twitches. Significant SSSEP blocking effects can be seen in intervals where the FPP is below the α-level of 1% (Bonferroni corrected). For left-hand twitches, significant SSSEP blocking was found in the interval 50–150 ms after twitch onset at channel FC4-CP4. For right-hand twitches, significant SSSEP blocking was found in the interval 150–200 ms after twitch onset at channel FC3-CP3. A full summary of significant results from all subjects can be found in Table 1B. Significant blocking intervals could be found in seven subjects at channels contralateral to the stimulated hand. These intervals were found between 50 and 250 ms after twitch onsets, with interval lengths in the range of 50–150 ms. On ipsilateral channels, no significant blocking intervals were found (not shown in Table 1).

### 3.4. BCI performance

The three-class BCI performance was evaluated by means of a 10 × 10 cross-validation when using SSSEP features, P300 features, or SSSEP blocking features. Table 1C summarizes the classification accuracies of all subjects. All classification results above the 1% chance level (Müller-Putz et al., 2008) are highlighted as bold values in the table. Accuracies better than random were found in 12 subjects using P300 features, in nine subjects using SSSEP features, and only in six subjects using blocking features. Mean and SD were computed only over subjects with accuracies significantly better than random. When comparing the accuracies of all three types of features one can see that the mean accuracies are in the range of 46.2 ± 1.8(SD)% for blocking feature classification, 48.6 ± 6.2(SD)% for SSSEP features, and 50.7 ± 8.5(SD)% for P300 features. Additionally, the hybrid BCI performance was computed when using the combined SSSEP, P300, and blocking features for classification, showing various results. In some subjects, an improvement in accuracy of the combined over the best single feature set could be found, whereas in some other subjects, no improvement or even a drop in performance to chance level could be observed. Classification accuracies better than random could be found in seven subjects, with a mean accuracy of 55.5 ± 8.5(SD)%. One subject, s10, did not reach accuracies above chance level using any feature set. This can be explained by the fact, that in s10, unexpectedly strong alpha waves were present in the EEG throughout all measurements, interfering with the actual features used for classification and leading to a rejection of around two thirds of the trials. Over all other subjects (s10 excluded), the mean rejection rate of trials due to artifacts was 7% using SSSEP features and 6% using blocking features. When using P300 features, no trials at all were rejected but the number of averaged segments per trial was reduced accordingly (on average, 6.997 ± 0.07(SD) averaged segments per hand and trial).

**Figure 12:**
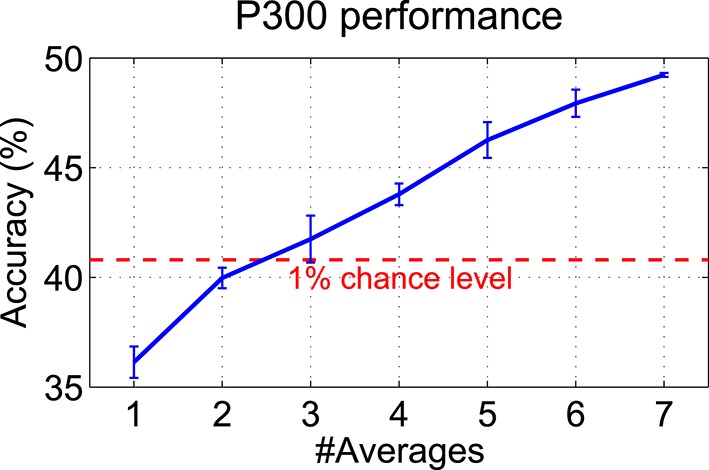
**Influence of the number of averages on the P300 performance**. The grand average of the classification accuracies (10 × 10 cross-validated) over all subjects is shown when averaging different numbers of twitch segments per trial (1–7). Random subsampling was used to select different numbers of twitches within trials. The whole procedure was repeated 10 times in order to get a more reliable estimate (mean ± SD are shown).

When extracting P300 features, all seven twitch segments per trial and hand were averaged. Figure 12 shows the grand average classification accuracies (10 × 10 cross-validated) for different numbers of averages ranging from one to seven. The P300 accuracy is monotonically increasing with the number of averages per trial from 36% (one segment only, i.e., no averaging) to 49% (seven segments averaged). The actual 1% chance level (Müller-Putz et al., 2008) of 40.8% is also shown in the figure.

## 4. Discussion

Within our work, the impact of transient target stimuli on the SSSEP was investigated in a real-life BCI setup. SSSEPs could successfully be evoked by simultaneous left and right index finger stimulation with repetitive tactile stimuli. Subject-specific stimulation frequencies were determined by means of a screening procedure in order to maximize the individual SSSEP responses. The overall screening results showed grand average tuning curves with peaks at 27 Hz, which is fully in line with previous findings by Müller et al. (2001). Moreover, the subject-specific stimulation frequencies which were selected after screening are in a similar range as the individual tuning curve maxima reported in previous studies (Breitwieser et al., 2011, 2012).

By embedding twiches at random positions into the repetitive stimulation signals, P300 potentials could successfully be evoked in addition to the steady-state response. The P300 component is usually defined as ERP with a positive deflection with a latency of about 300 ms (Farwell and Donchin, 1988). In our study, the grand average P300 response at channel Cz was found between 300 and 400 ms which is in line with this definition. Moreover, the latency and shape of the P300 component are fully in agreement with the results reported in other studies involving pure tactile P300 (Brouwer and van Erp, 2010; van der Waal et al., 2012). However, in our study, the P300 component was most prominent at central channel location above the somatosensory cortex, with an activation bilateral or contralateral to the stimulated hand, which was not reported in any other of these studies. Yet another transient response, namely a positive deflection with shorter latencies and at a more frontal location than the standard P300 response was found by Severens et al. (2013). These various results may be explained by the use of different tactile stimulators, stimulation patterns, and target body locations. In the study of Severens et al. (2013) for example, the index, middle, and ring finger tips were stimulated simultaneously per hand by means of Braille stimulators with complex stimulation characteristics involving different pins of the Braille stimulators. In contrast, in our study, we only used a single stimulator for each index finger tip but complex temporally modulated stimulation patterns. Moreover, due to the short inter-stimulus intervals between twitches, it is possible that some kind of overlapping ERPs instead of pure P300 potentials may have been evoked, which may explain the differences in our results. Some overlapping effects were already observed in the auditory domain using two concurrent tone streams with randomly appearing deviant tones (Pokorny et al., 2013).

The main focus of our work was to investigate the impact that twitches may have on the SSSEP. By means of a fast LAS feature extraction setting and statistical validation methods it could be shown that twitches have an attenuation effect on the SSSEP which usually cannot be captured with standard analysis methods. Significant SSSEP blocking effects time-locked to twitch positions were found in seven subjects. This shows that the attempt to combine different types of stimulation signals like repetitive signals and twitches has a mutual influence on each other. As demonstrated in an ideally simulated stimulation signal, this influence is originated at the level of stimulus generation but becomes apparent as physiological effect in the SSSEP. Similar results were also presented by Xu et al. (2014) who found out that SSVEPs and ERPs were not two absolutely independent features in their study. However, our significance test only proved the presence of significant attenuations of the SSSEP, but no conclusions about the actual blocking strength–full interruption or just attenuation of SSSEPs–can be made. Moreover, the exact positions of reported blocking intervals need not necessary coincide with real physiological effects. The reason for this are fundamental limitations in the simultaneous time-frequency resolution referred to as Gabor limit (Gabor, 1946) which prevents a more detailed characterization of the observed blocking effects in the time or frequency domain.

The general principle of an SSSEP-based BCI is that users intentionally modulate the SSSEP by focusing attention on one of the stimulated target locations (Müller-Putz et al., 2006). However, the SSSEP blocking effects found in our work may prevent subjects from effectively modulating the target SSSEP. According to Müller-Putz et al. (2006), time points of best class separability were generally reached only after a few seconds after cue onset. So, such time points of highest separability may have never been reached in our study due to repeated interruptions of the steady-state potential. We therefore investigated the information content about the target class that is contained in different feature sets by means of a classifier. In the SSSEP feature set, we wanted to reduce any transient effects and used the mean SSSEP strengths averaged over a long time interval of 7.5 s. In contrast, in the P300 feature set, short time segments after twitch onsets were used. Using a third feature set, we also investigated if SSSEP blocking effects may contain additional information useful for classification, similar as reported by Xu et al. (2013) in the visual domain.

When looking at the three-class BCI performance, classification accuracies significantly better than random could be reached by nine subjects using SSSEP features and by 12 subjects using P300 features respectively. The average classification accuracies (counting subjects with accuracies better than random only) were on similar performance levels, namely 49% for SSSEP features and 51% for P300 features. Using blocking features, accuracies significantly better than random could be reached only by six subjects, with an average performance of 46%. When using the combined feature set for classification, an improvement in accuracy could be found only in some subjects, whereas in others, no improvement or even a drop in performance to chance level could be observed. The main reason for this may be that the number of combined features (in the order of 1350) was simply too high compared to the number of trials (80 trials per class) so that even a shrinkage-based classifier could not extract all useful information any more. Moreover, the respective numbers of features within the combined feature set were highly different between SSSEP (2%), P300 (71%), and blocking (27%) features so that their relative importance may be biased toward P300 features. Further optimizations, such as reducing the total number of combined features and balancing their relative numbers in the combined feature set may therefore be required. So, unlike in the study of Xu et al. (2013), blocking features could not successfully be used to increase classification performance. However, they used a completely different BCI setup, namely a visual matrix speller with only one repetitive stimulation (flicker) frequency. In our work, blocking feature classification performance was above chance level only in subjects where SSSEP performance was also significant, and could never improve classification in cases where SSSEP classification was below chance level. This indicates that blocking features did not contain any additional information about the intended class but simply reflected SSSEP features extracted from shorter time intervals within the whole focus attention period. Moreover, a direct relationship between significant SSSEP blocking effects and reduced SSSEP accuracies could not be observed in our results. However, for blocking feature classification, all channels from SSSEP channel set were included whereas the blocking intervals reported in Table 1B only reflect significant results from two channels.

Even though accuracies better than random were reached by most subjects, the overall BCI performance was rather moderate and presumably hardly sufficient for communication purposes. The minimum performance level of 70% usually required for communication (Kübler et al., 2004) could not be reached by any of the subjects. However, this performance level was defined for a two-class BCI and, therefore, cannot be directly applied to our three-class BCI setup. The main reason for the rather moderate BCI performance could be that both types of brain signals—SSSEP and P300—cannot be evoked at the same time, since one is detrimental to the other. Also Severens et al. (2013) found no boost in performance when combining SSSEP and ERP features. On the one hand, the use of many twitches would be beneficial for P300, since as shown in Figure 12, averaging of many twitch segments increases the P300 accuracy. On the other hand, many twitches within short time may cause overlapping ERPs and many interruptions of the SSSEP, presumably lowering SSSEP and P300 performance. One obvious solution would be to increase the trial durations, so that many twitches could be embedded with large inter-stimulus intervals into the repetitive stimulation signal. The disadvantages of longer trial durations are of course lower information transfer rates and higher susceptibility to EEG artifacts. Another reasons for only moderate P300 performance may be that in some subjects a P300 was present not only after target but also after non-target twitches (not visible in the grand average response), possibly due to too strong (100% attenuation) twitches which drew attention toward the non-target hand. The same reason may also contribute to decreased SSSEP performance in some subjects, as non-target twitches may have prevented them from keeping attention focused on the target finger, thereby reducing attention modulation of the SSSEP (as opposed to SSSEP blocking effects which were caused by twitches irrespective of the target finger). As demonstrated by Adler et al. (2009), the distracting influence of non-target events in sustained somatosensory attention is mediated by perceptual load. In that study, distractors pulled attention toward to-be-ignored body locations in an easy detection task (low perceptual load), which was not the case in a challenging discrimination task (high perceptual load). Therefore, our results indicate that even though twitches were reported by most subjects as difficult to recognize and count, the perceptual load may have been too low since it was only a detection task. So, a more challenging discrimination task using different types of twitches could be beneficial in future. Moreover, in some subjects, only weak SSSEPs were present, even though individual stimulation frequencies were determined by a screening procedure. However, it is not yet known if there is some relationship between relative bandpowers from screening and SSSEP classification accuracies and if selecting the frequencies with highest bandpowers is even the best choice for strongest attention modulation effects.

## 5. Conclusion

Within our work, the role of transient target stimuli was investigated in an SSSEP-based BCI setup. Our findings suggest that different types of combined stimulation or brain signals such as SSSEP and P300 may not be regarded separately but have a mutual influence on each other. When designing a hybrid BCI based on SSSEPs and P300 potentials, one has to find an optimal tradeoff depending on the overall design goals or individual subjects' performance. Our results give therefore some new insights that may be useful for the successful design of hybrid BCIs.

## Author contributions

CP, CB, GM designed the study. CP, CB conducted all measurements. CP, CB analyzed the data. CP, CB, GM interpreted and discussed the results. CP wrote the manuscript.

### Conflict of interest statement

The authors declare that the research was conducted in the absence of any commercial or financial relationships that could be construed as a potential conflict of interest.
